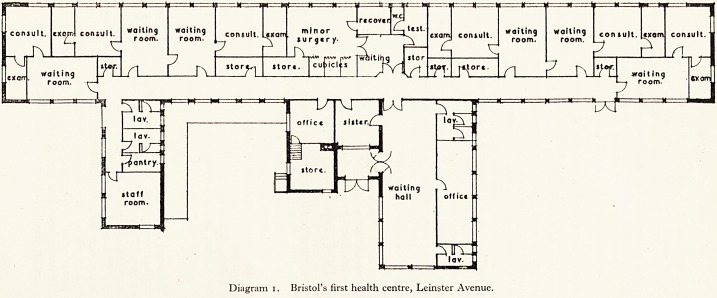# Bristol's New Health Centre

**Published:** 1952-04

**Authors:** R. C. Wofinden

**Affiliations:** Deputy Medical Officer of Health


### BRISTOL'S NEW HEALTH CENTRE
### in Leinster Avenue, Knowle West

BY

R. C. WOFINDEN, M.D., D.P.H.
*Deputy Medical Officer of Health*

One feature of the National Health Service Act, 1946, to receive almost unanimous approval from the medical profession, was the power given to local authorities to provide, equip and maintain health centres. So far no new health centre, built specifically for the purpose has been opened, and the health centre at Leinster Avenue which will come into operation this month (April 1952) will be the first. This is an account of the origin, nature and purpose of this centre.

The Knowle West estate in Bristol was built in the thirties, and although about 30,000 people live there, it possesses few community facilities and the medical services are inadequate. There is no resident doctor or dentist but there are two local authority clinics at the periphery. General medical practitioner services have been provided by doctors using, as sub-tenants, one room of a council house, the front garden of which has served the purpose of waiting room. The residents frequently asked the Council to provide better medical services.

The Health Committee tried to persuade general medical practitioners to *live* on the estate, but none of those who practised there were ever willing to do so. The Committee then considered supplying a resident doctor through the Poor Law Service, but found his work would have been limited to the destitute, so most of the population would still not have benefited. In 1939 two Corporation houses on the estate were adapted for the minor ailment treatment of patients of any age. These were staffed by nurses who also lived on the premises; this arrangement was valuable to the many residents who could not use a telephone when they wanted their doctor. The legality of this venture was open to doubt as it was claimed to be ancillary to the Southmead Municipal hospital which was situated some miles away. But this desperation measure proved to be an invaluable service, for many thousands of patients have been treated there each year. When the National Health Service Act came into force this service was continued by the District Nursing Association and, in due course, all its work will be transferred to the new health centre.

When the Act came into force, Knowle West was immediately considered to have a prior claim for a health centre, and negotiations were begun with a group of interested doctors and with the Ministry of Health. This is not the place to tell of all the frustrations and delays met in obtaining land in a built-up and established area, in persuading the Ministries of Health and Education that this was an urgent problem, in negotiating with all groups who might be interested such as the Executive Council, Local Medical, Dental and Pharmaceutical Committees, and the Regional Hospital Board; and in formulating plans which would receive the approval of all interested parties. Suffice it to say that ultimately it was agreed to provide a temporary health centre for the joint use of general medical practitioners and the local health and education authorities.

### THE BUILDING

The centre cost approximately £12,000 to build exclusive of land and internal equipment. It is of Orlit construction with a pleasing exterior and the planning is simple and restricted to imperative needs.

There are six self-contained suites for the use of the general medical practitioners; each suite is comprised of a consulting room, private waiting-room and examination room (with buzzer communication between the two former rooms).

There is a main waiting hall which is intended to be used when there is a child welfare clinic and when group health education is being undertaken. Opening off the hall is a large office in which all records will be stored, clerical and typing work undertaken, and telephone calls will be handled, and there is an inquiry hatch between the office and main hall.

The centre has a well-placed and reasonably large treatment room with recovery and waiting space annexes and the sister's room is nearby. In a separate wing of the building there is a staff room, kitchen and lavatory.

There is space enough to store small stocks of nursing appliances for use on the district. The centre is heated by gas-fired boilers and the separate rooms could be heated by electric fires if need be.

### EQUIPMENT

To begin with, only essential items have been provided; thus, for the personal use of the general practitioners, there are sphygmomanometers, various speculae, syringes, needles, etc., cupboards for instruments and drugs, and filing cabinets. The treatment room is equipped for minor operations, sight testing and examination of urines. There is also equipment for ante-natal, post-natal, child welfare and school sessions.

### STAFF

Considerable thought has been devoted to the staffing of the Centre for it is on this that its success or failure is likely to depend. The local authority will draft its own staff from its local pool; they will not be stationed at the centre. The personal staff for the general practitioners include two secretary-typists and one general clerk, all of whom may have to work morning and evenings with afternoons free.

There will be no health centre manager such as is advocated in the Report of the Health Centres Committee of the Central Health Services Council. Instead a superintendent nurse will be in general charge of the centre. She will have a deputy and there will be three full-time clinic nurses. Five nursing staff may seem rather generous but the treatment room, where they will work, will be open all day not only for the treatment of general practitioner patients but also for the minor ailment treatment of children referred from the local authority clinics. The superintendent will have to devote part of her time to administrative work; arrangements will have to allow for holiday and sickness relief. A night porter and part-time cleaners complete the establishment.

Diagram 1. Bristol's first health centre, Leinster Avenue.

The superintendent, her deputy and the clinic nurses will live in houses adjacent to the centre; the district nurses and midwives working in the district served by the centre will live in houses next to the clinic nurses. In this way it is hoped that the nursing staff will come to know one another better.

No residential accommodation has been provided for a caretaker.

### THE CENTRE IN OPERATION

Practitioners will use the Centre in the mornings and evenings for their usual " surgeries ". The local authority will use the premises, including the general practitioner suites, in the afternoons.

A general practitioner's patients calling at the inquiry desk will be conducted to his waiting-room and their records will be placed on the desk in his consulting room. Patients referred to the treatment room will wait in the annexe. Doctors' letters, home visit appointments made by the Centre, and patients' appointments at hospitals will be dealt with by the secretary-typists.

For the time being, no financial arrangements have been made for group practice because some of the doctors are already in partnership with doctors not participating in the health centre. New patients, not on any existing list and with no preference for a particular doctor will be shared fairly between the doctors. This allocation of new patients will be reviewed every three months.

For the time being, each practitioner will be responsible for his own night calls, holiday and sickness relief. Each practitioner will be responsible for the ante-natal care of his own patients and has agreed to give a medical opinion on school children referred to him by the school health service.

Health visitors, home nurses and midwives who work in the district will use the centre as their base; this will enable them to collaborate more closely with the doctors.

The doctors, together with the Medical Officer of Health or his representative and the Nursing Superintendent will form the Health Centre House Committee to settle domestic problems. Policy matters will be referred to a Joint Advisory Committee composed of representatives from the Local Health Authority, Executive Council and Local Medical Committee. This Joint Advisory Committee has no executive powers, but will make recommendations to its parent bodies. These two committees have been in action for six months already; the former, as the *working committee*, has worked out the details of organization.

### DISCUSSION

It would be easy for the critic to point out how far this venture falls short of the true health centre concept. There is no dental unit, pharmacy, X-ray or side-room laboratory. The local authority is supposed to provide these facilities, but there is no point in doing so if they will not be used! Neither the Local Dental nor the Local Pharmaceutical Committee wanted dental or dispensing facilities and the doctors were not anxious to establish a hospital out-patient department.

Initially, there will not even be a group partnership; indeed, there is no arrangement for mutual relief for emergency calls, or to cover holidays or sickness. But it should be remembered that the circumstances under which the centre has evolved are rather peculiar. They were certainly not envisaged by the planners who seemed to be under the impression that in established areas general practitioners might be willing to break existing partnerships and form new associations. Except in new towns I think there is small likelihood of full-time health centre practice being conducted by a group of doctors working as a firm. I doubt whether such an arrangement can be made for a completely new housing estate, for even here the new tenants probably already have a doctor, who can, if he wishes, follow his patient to the new area. I feel that the only chance of making such an arrangement will depend on the presence in any locality of a group of doctors who are imbued with patience, enthusiasm and courage, and who are prepared to take the initiative by making known their requirements to the Executive Council and Local Health Authority.

Certainly it is only the tenacity and enthusiasm of the doctors at Leinster Avenue which has got us thus far. Maybe in due course they will come closer together in their business and working arrangements. Perhaps X-ray and side-room laboratory facilities, a pharmacy and a dental unit will be added at a later date. In the meantime the venture will enable the general practitioners and health authority staff to see something of each other's work with its difficulties, triumphs and disappointments. Even our severest critic could hardly condemn such a desirable objective.

My doubts about the future are based, not on the difficulties of making personal adjustments in the interests of a better health service, but on economic considerations. The maintenance costs of this temporary centre will be nearly £200 a week, and of this only one third will be for local health and education authority services. The Ministry of Health has made no firm ruling on how much rent the doctors, through the Executive Council, will have to pay. So it seems that almost the whole of the cost may fall on the Local Health Authority (although this is subject to a Government grant of roughly fifty per cent). We need about thirty health centres to cover the whole of Bristol!

This article is based on details given in the Medical Officer of Health's Annual Report of the City and County of Bristol.

## Figures and Tables

**Diagram 1. f1:**